# Chemical Compositions of Metals in Bhasmas and Tibetan Zuotai Are a Major Determinant of Their Therapeutic Effects and Toxicity

**DOI:** 10.1155/2019/1697804

**Published:** 2019-01-10

**Authors:** Jie Liu, Feng Zhang, Velagapudi Ravikanth, Olumayokun A. Olajide, Cen Li, Li-Xin Wei

**Affiliations:** ^1^Key Lab for Basic Pharmacology of Ministry of Education and Joint International Research Laboratory of Ethnomedicine, Zunyi Medical University, Zunyi, China; ^2^Department of Pharmacy, School of Applied Sciences, University of Huddersfield, Queensgate, Huddersfield, West Yorkshire HD1 3DH, UK; ^3^Qinghai Key Laboratory of Tibetan Medicine Pharmacology and Safety Evaluation Northwest Institute of Plateau Biology, Chinese Academy of Sciences, Xining 810008, China

## Abstract

Minerals are alchemically processed as Bhasmas in Ayurvedic medicines or as Zuotai in Tibetan medicines. Ayurveda is a knowledge system of longevity and considers the mineral elixir made from “nature” capable of giving humans perpetual life. Herbo-metallic preparations have a long history in the treatment of various diseases in India, China, and around the world. Their disposition, pharmacology, efficacy, and safety require scientific evaluation. This review discusses the Bhasmas in Ayurvedic medicines and Zuotai in Tibetan medicines for their occurrence, bioaccessibility, therapeutic use, pharmacology, toxicity, and research perspectives. A literature search on Mineral, Bhasma, Ayurvedic medicine, Zuotai, Tibetan medicine, and Metals/metalloids from PubMed, Google and other sources was carried out, and the relevant papers on their traditional use, pharmacology, and toxicity were selected and analyzed. Minerals are processed to form Bhasma or Zuotai to alter their physiochemical properties distinguishing them from environmental metals. The metals found in Ayurveda are mainly from the intentional addition in the form of Bhasma or Zuotai. Bhasma and Zuotai are often used in combination with other herbals and/or animal-based products as mixtures. The advanced technologies are now utilized to characterize herbo-metallic preparations as Quality Assurance/Quality Control. The bioaccessibility, absorption, distribution, metabolism, and elimination of herbo-metallic preparations are different from environmental metals. The pharmacological basis of Bhasma in Ayurveda and Zuotai in Tibetan medicines and their interactions with drugs require scientific research. Although the toxic potentials of Bhasma and Zuotai differ from environmental metals, the metal poisoning case reports, especially lead (Pb), mercury (Hg), and arsenic (As) from inappropriate use of traditional medicines, are increasing, and pharmacovigilance is desired. In risk assessment, chemical forms of metals in Bhasma and Zuotai should be considered for their disposition, efficacy, and toxicity.

## 1. Introduction

Ayurveda is a knowledge system of longevity, and considers the mineral elixir made from “nature” capable of giving humans perpetual life [1]. Bhasmas are unique Ayurvedic metallic preparations. In the Ayurvedic Pharmacopoeia of India, mineral use accounts for 8%. These include Suvarna (Au), Rajata (Ag), Tamara (Cu), Lohamala (Fe), Manahshila (realgar, As), Kamksi (Al), Sudhu (Lime), Sisa (Pb), etc. [2]. Minerals have been used in traditional medicines since ancient times and are still in use today. For example, Lead (Pb)-based traditional medicines can be found not only in Ayurveda, but also in traditional Chinese medicines, traditional medicines used in Mexican, Pakistan, Iran, and Kuwait [3]. Mercury-based traditional medicines can be found in traditional Ayurveda, Siddha, Sri Lanka, Tibetan, and Chinese medicines [4–6]. Similar to Bhasmas, Zuotai, a mineral mixture, is included in hundreds of Tibetan medicines [7–9]. Thus, the existence of metals in some of the Asian traditional medicines is a reality.

Minerals (HgS, As_4_S_4_, PbS, PbO, etc.) used in Ayurveda and Tibetan medicines undergo extensive processing procedures like alchemy to alter their chemical forms in Bhasmas or Zuotai preparation, which are different from environmental metal forms (HgCl_2_, MeHg, NaAsO_2_, NaH_2_AsO_4_, Pb(CH_3_COO)_2_, etc.) and are the only suitable forms for oral medication. An understanding of the processed metal forms used in Ayurveda and Tibetan medicines and their Quality Assurance/Quality Control (QA/QC) is essential. Minerals/metals are not used alone, rather as the polyherbal-animal-metallic preparations in Ayurvedic and Tibetan medicines [2, 10, 11]. The herb-metal interactions are believed to assist the delivery of drugs to the target, contribute to therapeutic effects, and reduce toxicity. A discussion of the mixtures rather than individual metal provides a different point of view.

The traditional medicines are often perceived as “safe” because of their basis on plants or “natural” ingredients, and because they have been used for thousands of years in different cultural settings. However, risks can be associated with the use of mineral/metal containing medications, especially in self-care practices [12]. Heavy metal toxicity case reports from inappropriate use of these remedies are increasing in recent years, especially from Pb, followed by Hg and arsenic (As) [13, 14], and the pharmacovigilance is desired. The beneficial effects of any medicine often go hand-in-hand with toxicity. Even essential metals will become toxic with increasing exposure. A balance of their efficacy and toxicity is important.

This minireview started with mineral processing like “alchemy” to produce forms of metals suitable for oral medication, followed by their Quality Assurance/Quality Control (QA/QC). Pharmacokinetics on Pb, Hg, and As highlight that chemical forms of metals make a difference. The therapeutic effects and pharmacology were discussed, followed by differential toxicity to argue against the use of total metal content for their safety assessment. Finally, research perspectives were briefed.

## 2. The Preparation Procedures of Minerals in Ayurvedic and Tibetan Medicines Make a Difference

### 2.1. Bhasmas Preparation in Ayurvedic Medicine

In Ayurvedic medicines sold on the Internet, 20% contain excess metals [13]. In fact, some experts estimate that 35-40% of Ayurvedic medicines contain at least one metal [15] in the form of Bhasmas. Bhasmas are unique Ayurvedic metallic/minerals preparations, the minerals were treated with herbal juice or decoction and animal-based products such as horns, shells, feathers, milk and urine, and further subjected to repeated incinerations. The resultant Bhasmas are called Parada (Hg), Lauha (Fe), Swana (Au), Rijata (Ag), Tamra (Cu), Naga (Pb), Yasada (Zn), and Vanga (Sn), as well as Adbraka (mica) [16, 17]. This process of incineration and addition of medicinal herbs and animal-based products is believed to remove impurities and eliminate the harmful components of herbo-metallic preparations [7, 18]. The particle size of these processed metals usually becomes smaller, so called “ancient nanomedicine” [17, 19, 20].

Table 1 provides examples of “standard procedures” for these metallic preparations. For example, Ayurveda utilizes Rasashastra which is a process of imparting the medicinal value of minerals in Ayurveda. In Rasashasra there are many methods to control and convert mercury to “Rasasindura” which is beneficial and less toxic than inorganic mercury such as HgCl_2_ or organic mercury such as methylmercury (MeHg) [4]. The procedures for preparation used Swarna (gold), Parada (mercury) and Gandhaka (sulfur) in different ratios [21]. The standard procedure for preparation of Lead Bhasma (Naga) could remove many impurities and produce PbS and PbO [3]. The standard procedure of (Cu) could produce CuS [22]. The processing procedures for iron formulations (Lauha) are important for iron efficacy and safety [23]. The Ayurvedic medicine Tarakeswara Rasa is a metal mixture, and XRD analysis revealed that it contains Fe_2_O_3_ (maghemite) in major phase, and SnO_2_ (cassiterite), HgS, SiO_2_, and HgO in minor phases [24]. The preparation of Trivanga Bhasma (Sn, Zn and Pb), examined by XRD analysis, shows crystalline nature and nanosized particles by Scherrer's equation, and by SEM analysis, lead, zinc and tin oxides show well-defined plate-like structures [25]. The standard preparation procedures for Rajata Bhasma (Ag) and silver nanoparticles are detailed [26]. Yashada (Zn) preparation and its antidiabetic role were also documented [19]. The Jasas Bhasma (ZnO) preparation and toxicity profiles were examined in mice [27]. The synthesis of Vanga Bhasma by electric muffle furnace produced 100% nanoparticles (50-100 nm range) and was characterized by modern techniques such as TEM, SEM, EDX, XRD, DLS, and FTIR [28].

The Bhasma preparation could make a big difference in toxicity. For example, Naga Bhasma contains Pb, and undergoes several stages of the preparation. In a study comparing the effects of various preparing stages of Naga Bhasma and pure Pb used in the Naga Bhasma production, pure lead-treated animals showed a deficit in learning and memory, evidenced by spending more time in the dark compartment in passive avoidance test. However, animals treated with the stage 1 to 4 Naga Bhasma showed a gradual increase in the memory and learning, consistent with histopathology of the Cornu Ammonis (CA) region of the hippocampus [34]. Another example is the Tamra Bhasma (incinerated copper) prepared with and without* Amritikarana*, a procedure by mixing with purified sulfur and juice of* C. jambhiri* Lush, kept in the corm of* Amorphophallus campanulatus* Linn, and subjected to heat treatment. The toxicity potentials between two preparations were quite different [35].* Shodhana, Bhavana, *and* Marana* are the steps involved in Bhasma preparation of metals.* Shodhana* detoxifies and makes material suitable for* Marana* [36]. The role of* Shodhana *is the key step in producing Bhasmas [37], the toxicity of Tamra Bhasma is markedly reduced with* Shodhana *[38].In Pravel Bhasma preparation, the raw material Coral calx contains CaO_3_, but in the final product, CaO was identified and the particle size was smaller [29]. Biotite mica as raw materials may be harmful when used directly, as they carry considerably high amounts of trace-elements that can cause undesirable effects. Abhrak Bhasma (mica ash) processing is essential to remove these toxic factors. Purification steps influence the structural distortion while heating and quenching can form nanosized particles. At the same time, the toxic elements are leached out from mica to the quenching media through an ion exchange process [30].

### 2.2. Zuotai Preparation in Tibetan Medicine

Like Bhasmas in Ayurveda, Zuotai, a mixture of metal ash, is included in many famous Tibetan medicines [8]. Zuotai also undergoes the similar, tedious processing procedures as Bhasmas [7, 8], which usually takes 3-4 months of repeated treatment and incineration procedures using herbals and animal-based products [7, 8].

Zuotai is composed of Nengchi Bakuang and Nengchi Bajin ashes, and the XRD analysis revealed that Nengchi Bakuang contains not only the major chemical components from mica such as SiO_2_, CaCO_3_, K_2_Ca(SO4)_2_ H_2_O, and KCl, but also metals such as FeAs_2_, FeAs, Fe_2_As, Cu_2_S, AsFe and so on [32]. In NengchiBajin used to refine Zuotai, the XRD analysis revealed AuPb_2_, PbO, PbSO_4_, Ag_2_S, CuS, SiO_2_, CuO, FeS, SnS, and other structures in different metal ashes [33]. The major metal-compound of Zuotai is metacinnabar (54% of *β*-HgS) [31].

In traditional Chinese medicines, cinnabar (96% of *α*-HgS) is subjected to extensive grinding and washing (called* Shui-Fei*) for at least 3-4 times to remove impurities, and this procedure is very important for the safe use of cinnabar in traditional medicines [11, 39].  Table 2 compared the similarities and differences of processed *α*-HgS and *β*-HgS and their inclusion in Tibetan medicines, Chinese medicines, and Ayurvedic medicines.

To characterize Zuotai (gTsothal) used in Tibetan medicine, the energy dispersive spectrometry of X-ray (EDX), scanning electron microscopy (SEM), atomic force microscopy (AFM), and powder X-ray diffraction (XRD) were used to assay the elements, micromorphology, and phase composition of nine Zuotai samples from different regions, respectively. EDX result shows that Zuotai contains Hg, S, O, Fe, Al, Cu, and other elements. SEM and AFM observations suggest that Zuotai is a kind of ancient nanodrug. Its particles are mainly in the range of 100-800 nm, which commonly further aggregate into 1-30 *μ*m loosely amorphous particles. XRD test shows that *β*-HgS, S8, and *α*-HgS are its main phase compositions [31].

### 2.3. Quality Assurance/Quality Control (QA/QC) of Bhasmas and Zuotai

The advanced technologies such as EDX, XRD, AFM, and SEM as mentioned above were utilized to characterized Bhasma preparations. In addition, Inductively coupled plasma mass spectrometry (ICP-MS), Fourier transform infrared spectroscopy (FTIR), Scanning electron microscopy with energy dispersive X-ray spectroscopy (SEM-EDX),Gold amalgam enrichment-atomic fluorescence spectrometry (GAE-AFS), Raman spectrum and particle size analysis, etc., have now been used to characterize Ayurvedic Bhasma preparations and Tibetan preparation Zoutai [22, 25, 40]. Characterizing the physicochemical properties of Bhasmas and/or Zoutai in herbo-metallic preparations is a prerequisite in uncovering the mysteries of these ancient remedies. For example, Suvarna Bhasma Parada Maritwas characterized by XRD, SEM, energy dispersive X-ray analysis (EDAX), laser particle size distribution (PSD) analysis, FTIR, atomic absorption spectroscopy (AAS), and physicochemical parameters, such as the loss on drying (LOD), loss on ignition (LOI), and acid insoluble ash (AIA) [41].* Shodhana *procedure leads in the formation of bonds between surface particles of Tamra and* Shodhana* media by Fourier transform infrared (FTIR) spectroscopy [42]. Vanga Bhasma was characterized by TEM, SEM, EDX, XRD, DLS and FTIR [28]. An understanding of the processed metal forms in Bhasmas and Zuotai and their QA/QC procedures are essential to evaluate their efficacy and toxicity.

## 3. Chemical Compositions of Pb, Hg, and As Affect Their Bioaccessibility and Disposition in the Body

### 3.1. Lead (Pb)

The speciation and bioavailability of lead in a dozen of Ayurveda and/or traditional medicines were analyzed. Speciation of Pb was performed using a sequential fraction and Extended X-ray Absorption Fine Structures (EXAFS), and Pb bioavailability using a physiologically based in vitro extraction tests. The results revealed that inorganic-bound Pb species dominated in Ayurveda, with various Pb species [43]. A physiologically based extraction test was used to assess the bioaccessibility of Pb and As in Ayurveda [44]. The test consisted of a gastric phase at pH 1.8 containing organic acids, pepsin and salt, followed by an intestinal phase, at pH 7 and containing bile and pancreatin. Consumption at recommended doses of Pb and As-containing Ayurvedic medicines resulted in higher bioaccessibility of Pb or As, leading to the exceedance of the standard for acceptable daily intake of toxic elements. For example,* Mahayograj Guggulu* that had been compounded with Bhasmas (calcined minerals),including Naga Bhasma, resulted in a very high amount of Pb (52,000 mg/kg, 1,000-fold higher than others) with 100% bioaccessibility [44].This could be the reason that inappropriate use of* Mahayograj Guggulu* is the most common cause responsible for Pb intoxication cases.

### 3.2. Mercury (Hg)

Absorption of cinnabar (HgS, 0.2%) from the gastrointestinal tract is much less than mercuric chloride (7–15%) and methyl mercury (95%), while the metallic mercury is almost unabsorbed (0.01%) from the gastrointestinal tract [39].

The Ayurvedic medicine* Mahayograj Guggulu *also contains a high amount of bioaccessible *α*-HgS (cinnabar, 25,500 mg/kg), while* Arogyavardhini Vati* contains the high amount of bioaccessible *β*-HgS (metacinnabar, 13,050 mg/kg).The bioaccessibility of HgS could be enhanced with increasing dissolved organic carbon content, as revealed by Fed Organic Estimation human Simulation Test (FOREhST) that measures bioaccessibility in humans [45, 46].Under mimetic intestinal and gastric conditions, the chemical components dissolved from HgS are analyzed by infrared spectroscopy and Raman spectroscopy, with mercuric polysulfide (i.e., HgS_2_(OH)− and Hg_3_S_2_Cl_2_) as major dissolved chemical forms [46]. The artificial intestinal juice containing L-Cys or GSH could facilitate the release of Hg from HgS [47]. Nonetheless, dissolved mercury existed in the investigated Ayurvedic medicines had low (<5%) bioaccessibility, that could explain the low Hg risk in these preparations [45].

Biotransformation occurs for mercury, and HgS can be biotransformed to Hg^2+^ in the gut [4, 39]. However, there was a notion that cinnabar might be transformed to methylmercury by gut microbiota as methylmercury is produced in natural environments from inorganic mercury by anaerobic bacteria, which is not true [39]. As in the human gut, no evidence for cinnabar to be converted into methylmercury is found [48]. Recent studies found that two-gene clusters, hgcA and hgcB in microbes, are required for mercury methylation [49], and by directly measuring MeHg production in several bacterial and archaeal strains encoding hgcAB, the possessing of hgcAB could predict Hg methylation capability [50], and the capacity of gut* E. coli* to produce methylmercury is very low (4, 000 times lower than hgcAB*-*encoding* D. desulfuricans* ND132), close to zero [49].

### 3.3. Arsenic (As)

Arsenic toxicity is highly dependent on the chemical forms. For example, the acute oral LD_50_ values for sodium arsenite (As^3+^, 15 mg/kg), sodium arsenate (As^5+^, 115 mg/kg), realgar (As_4_S_4_, 3.2 g/kg), and arsenobetaine (AsB, 10 g/kg) are hundreds or thousands-fold different [51].

Tissue metal accumulation represents the outcome of metal disposition in the body and the resultant cellular load. Arsenic is subjected to methylation metabolism, and the methylation capacity greatly affected their toxicity potential. The average total arsenic concentration ina Niu-Huang-Jie-Du Pian (NHJD) is approximately 7% (i.e., 70,000 ppm), corresponding to 28 mg of arsenic per pill, of which only 1 mg of arsenic finds its way into the blood stream, and 40% of this absorbed arsenic (0.4 mg) is excreted in urine. Realgar exposure results in various arsenical metabolites in the urine, including MMA, DMA, arsenobetaine, and an unknown metabolite [52]. Bioaccessibility of sodium arsenate and sodium arsenite (80-85%) is quite high as compared to realgar (4%). On the other hand, in* Mahayograj Guggulu,* arsenic is more bioaccessible (about 50%), probably due to a large proportion of oxidized arsenic [44].In rats treated with Liu-Shen-Wan (As 7.7-9.1%) and NHJD, poor bioavailability of As and Hg from TCM as indicated by low relative bioavailability of As (0.60-1.10%) and high levels of As were excreted in feces [53].

Determination of* in vitro *bioaccessibility of metals in traditional medicines is the prerequisites for us to understand their body fates. The bioaccessibility could affect the ability of Pb, Hg, As, and Cd to enter cultured cells [54], as well as in animals [43]. Bioaccessible Hg contents of 29 HgS-containing traditional medicines were determined by cold-vapor atomic fluorescence spectrometry after in vitro extractions with the simulated gastrointestinal fluids. According to the daily dosages, the bioaccessible Hg exposures of the majority (27/29) of TCMs except for Shusha Anshen pills and Zixue Pills were within the permitted daily exposure limits established by the International Council for Harmonisation, demonstrating that these traditional medicines may be safe when administrated following the appropriate instructions [55].

Many of the minerals/processed metals are not water soluble. The questions are raised regarding how they get into cells. A recent study on Swama Bhasma (gold) and chemically synthesized gold particles in cells found there are two models for these particles to get into the cells, one is micropinocytosis, and another one is clathrin-dependent receptor-mediated endocytosis [56]. Bhasmas could be called ancient nanodrug, to study its uptake into the cells, and/or interaction with cell surface molecules could help our understanding of these ancient nanoparticles.

The sulfide forms of metals usually had low bioaccessibility, lower absorption rate, so it is no surprise that their toxicity potentials are lower compared to environmental chemicals. How do they produce biological effects? Recently, gut microbiota emerges as the hot area of research. “Gut-liver”, “gut-brain-microbiota” axis has been proposed, and modulation of gut microbiota represents new therapeutic approaches [57]. In this regard, we have found Zuotai in Tibetan medicines could affect gut microbiota as a means of its biological effects (manuscript in preparation), and more studies are warranted in this emerging area.

Thus, chemical forms of metals in herbo-metallic preparations are an important determinant in assessing their bioavailability and their fate in the body.

## 4. Chemical Compositions of Metals Determine Therapeutic Efficacy and Pharmacological Effects in Ayurvedic and Tibetan Medicines

### 4.1. Therapeutic Efficacy and Safety Are Most Important

Traditional remedies can survive thousands of years because of their efficacy and safety in patients. Listed below are a few examples:* Arogyavardhini Vati* is a herbo-metallic preparation containing parade (HgS), Tamra (Cu), Abhra (Mica), Loha (Fe) and other 7 herbs, and has been shown to be effective against Triton WR 1339 induced hyperlipidemia in rats [58]. In a clinical trial, all patients received Arjuna powder (5 g, twice a day) for the first 3 weeks followed by* Arogyavardhini Vati* (500 mg, twice a day) for 4 weeks, and the satisfactory efficacy was observed with tolerable side effects and toxicity [59]. The levels of total cholesterol, LDL-cholesterol, and triglyceride were decreased, while the HDL-cholesterol was increased, with a reduction in blood glucose and C-creative protein. The serology for liver and kidney parameters were in normal range [59].* Arogyavardhini Vati* together with other herbs was also effective in the treatment of a life-threatening skin emergency erythroderma [60].

There are many formulations in Ayurveda against diabetes. For example, antidiabetic formulations of Naga Bhasma were discovered 900 years ago, with 44 formulations of Naga Bhasma were developed, and herbs and animal-based products enhance the antidiabetic action, prevent diabetic complications, and reduce side effects [61]. Jasada Bhasma (zinc ash) [62], Trivanga Bhasma [25],* Tarakeswara Rasa* [24], and gold- and mercury-containing preparation,* Shadguna Balijarita Makaradhwaja* [63] have been reported to be effective for treating diabetes mellitus clinically. In a randomized controlled study in iron deficiency anemia patients,* Kasīsa Bhasma* was effective in the treatment of the iron deficiency [64].

Tibetan medicine is one of the important medical heritages of the world [65]. A systematic review of 39 clinical studies on Tibetan medicines in the west indicated that more pharmacology and clinical studies are needed [66]. In Tibetan medicines used for the liver diseases, 193 recipes (including 181 plants, 7 animal products, and 5 minerals) were used [67]. For example, Zuotai (5.81%)-containing* 70W Zhen-Zhu Wan* (also called* Rannasangpei, *Padma-28) is effective clinically in the treatment of liver diseases [67], and vascular dementia [68]. Mice received* 70W-Zhen-Zhu Wan* in a 7-day study did not show overt toxicity, but dose-dependently protected against CCl_4_-induced liver injury by inducing Nrf2 and Nrf2 target genes [69]. It should be noted that Induction of Nrf2 antioxidant system offers the generalized immunomodulation and protection not only in the liver but also in the brain [70].* 70W Zhen-Zhu Wan* improves bilateral common carotid artery occlusion-induced learning and memory deficit in rats, along with decreased oxidative stress and enhanced Nrf2 targeted antioxidant components ([68]).

Brag-zhun is another example included in Tibetan medicines. In the chemical analysis of 13 catches of Brag-zhun, 26 kinds of mineral elements were found in a total of 3%, organic matter ranged from 29%-71%, acid insoluble ash 2%-39%; and the water soluble extract was 28%-57% [71]. These minerals are clinically safe at appropriate dosages. For example, in a clinical trial, Zuotai (10%) containing* Dangzuo *was given to patients at the clinical dose (6.7 mg/kg/day) and duration of 30 days, and their serum biochemical indicators, blood routine indicators and urine routine indicators showed no significant adverse changes [72]. Zuotai (5.81%) containing* 70 Wei-Zhen-Zhu Wan* (*Ranansongpei*) was given to 123 patients at the clinical dose (1 g/day for 15 days), and no apparent adverse effects were observed. The mercury content in serum and urine was increased (~40-90%), and the majority of mercury was found in feces (~28-37-fold). Serum mercury levels returned to normal after stopping medication (Yang et al., manuscript submitted). In a cross-sectional study, 50 patients taking Zuotai-containing Tibetan medicines (130 *μ*g/kg/day versus RfD of 0.3 *μ*g/kg/day) for average 7-month exposure and compared to 50 patients taking the same drug but without Zuotai. Results showed that patients taking Zuotai-containing Tibetan medicines did not show mercury toxicity to the liver and kidney, with normal neurological, cardiovascular and dental findings, suggesting that Zuotai-containing Tibetan medicines at clinical doses do not have appreciable adverse effects clinically and may exert a possible beneficial effect on neurocognitive function [73].

Ayurvedic and Tibetan medicines have thousands of years practice, based on clinical efficacy and safety. However, detailed publications on well-designed clinical trials are not easily found. Padma Inc. in Switzerland has been producing selected complex formulas such as Padma-28 (70W) from Tibetan medicine for 40 years, with satisfactory clinical therapeutic effects and safety. By complying with QA/QC standards and requirements of regulatory agencies, the quality, efficacy, and safety are ensured [74].The patient surveillance and biomarker detection are desirable when taking Bhasmas [75]. The personalized medication is important to balance benefits and risks.

### 4.2. Pharmacological Studies on Herbo-Metallic Preparations

Accumulating pharmacological studies on Ayurvedic and Tibetan medicines would help our understanding of the beneficial effects of these traditional remedies. For example, Vasant Kusumakar Ras (VKR), which contains heavy metals (Pb, Hg, Au, and Fe) and polyherbs, is more effective than individual ingredients in the treatment of diabetes [76], as the metals are in the form of Bhasmas, which is different from environmental metals. Gold- and mercury-containing preparation,* Shadguna Balijarita Makaradhwaja* is effective against streptozotocin-induced diabetes in rats [63]. Zuotai has a strong ability to ameliorate depressive-like behaviors in chronic unpredictable mild stress -treated mice through inhibition of the hypothalamic-pituitary-adrenal axis and upregulation of monoamine neurotransmitter [77]. In mice treated with HgCl_2_ (10 mg/kg) and 10-fold higher HgS (100 mg/kg) or cinnabar for 10 days, the Hg concentrations in the brain, serum, liver, and kidney of HgCl_2_-treated mice were 15-, 20, 65- and 87-fold higher than HgS groups, respectively. Most importantly, only HgS could significantly decrease brain serotonin levels, whereas HgCl_2_ was ineffective [78].

Many metallic preparations had antibacterial activity. For example, Rajata (Ag) Bhasma nanoparticles could suppress* Staphylococcus aureus, Escherichia coli*,* Pseudomonas aeruginosa*, and* Enterococcus faecalis* [26]. Yashada Bhasma (Zinc clax) could inhibit* Propionibacterium acne* and suppresses acne induced inflammation in vitro [79]. Tamra (Cu) Bhasma is effective in inhibiting the growth of gram-negative (*P. aeruginosa, K. pneumoniae*) and gram-positive (*S. aureus*) bacteria [36]. The pharmacological basis of Bhasmas and Zuotai is increasingly explored.

### 4.3. General Hypothesis for Bhasmas and Zuotai to Produce Beneficial Effects

**Hormesis **refers to a phenomenon in which low doses of a chemical are beneficial, while high doses are toxic [80]. Even the toxic heavy metals can be beneficial to humans at the low concentrations, exerting activities apart from its toxicity. The Indo-Tibetan tradition claims that proficiency in the suggested longevity practices of meditation, diet, and physical exercise (yoga), will result in profound antiaging, stress-mediating and health-enhancing effects through modulation of the body's protective and regulatory systems. Processed metals such as Au, Hg, Pt, Pb, and Fe are included in some homeopathic remedies, and these remedies usually have broader healthy properties than homeopathic therapy alone [81]. Bhasmas and Zuotai are not used alone, rather as additions to herbo-metallic mixtures in very small amounts. The low dose of the processed minerals would fit, at least in part, into the hormesis theory. For instance, Ayurvedic medicine* Oxidard* at the low dose is better than the high dose in protecting against chronic stress [82].

**Adaptation **is a generalized mechanism for traditional medicines. The herbo-metallic mixtures could be envisioned as a whole, and “Herbogenomic” [83] would be a useful approach to identify the key molecular pathways following the administration of herbo-metallic mixtures at different dose levels. These herbo-metallic preparations could increase the immune function, induce antioxidant pathways, or affect drug-processing genes in a way similar to “program the liver” [84] to produce beneficial effects. Listed below are some, but not all, possible adaptation mechanisms:

1. *Induction of the Nrf2 antioxidant pathways.* Many Ayurvedic [82] and Tibetan medicines [85] could activate the Nrf2-antioxidant pathway to reduce oxidative stress, as the activation of Nrf2 is a universal protective means against toxic stimuli [86].
2. *Immunomodulation and anti-inflammatory properties. *Ayurvedic medicines like Jasad Bhasma [27] and Zuotai-containing Tibetan medicines [67] could modulate immune functions to exert beneficial effects and anti-inflammatory actions.
3. *Modulation of metabolism. *Diabetes and hyperlipidemia are common metabolic disorders. Zn-based Jasada Bhasma [62], gold-containing preparation* Shadguna Balijarita Makaradhwaja* [63] and lead-based Naga Bhasma [3, 61] were reported to be effective against these metabolic disorders. The effects of herbo-metallic preparations on metabolism process and drug-processing gene expression are an open-field of investigation
4. *Modulation of neurological function*. The Tibetan medicine Zuotai is effective in ameliorating depressive-like symptoms in chronic unpredictable mild stressed mice by increasing brain 5-HT levels [77]. Gold preparations (Swarna Bhasma and* Unani Kushta Tila Kalan*) restored restraint stress-induced elevation in levels of the brain catecholamines (norepinephrine, epinephrine and dopamine) and 5-HT to produce beneficial effects [87].
5. *Tissue repair and regeneration. *Gold nanoparticles could perpetuate “stemness" to enhance self-renewal and pluripotency [88], the formulations containing gold [89] along with* Shorea robusta* resin and flax seed oil for local application showed significantly better wound healing activity. How do Bhasmas and Zuotai-containing traditional remedies promote tissue repair are important issues and warrants further investigation.

**Use poisons to attack poison **is a philosophy in traditional Asian medicines. It should be borne in mind that pathological (diseased) condition is quite different from physiological conditions. For refractory diseases and malignant diseases, “using a poison to attack another poison” is a traditional strategy to save a life from cancer, and the use of toxicants like arsenic to kill cancer cells is well justified [90]. In face of brain emergency, such as stroke, trauma, brain bleeding and infarction, HgS-containing recipes were effective in keeping conscious. Under such pathological conditions, the efficacy of herbo-metallic preparations overweighs their toxicity [91].

## 5. Chemical Forms of Metals Are a Major Determinant to Their Toxicity

Any substance is potentially toxic. In risk assessment, the total metal content was used for risk assessment. For example, PbS and PbO are frequently taken as Pb acetate, HgS is frequently taken as HgCl_2_, and As_4_S_4_ is frequently taken as NaAsO_2_. However, their toxic potentials are quite different (Table 3).

### 5.1. Cytotoxicity in Cultured Cells

In cell cultures, there is over a 200-fold difference in cytotoxicity between cinnabar (HgS) and HgCl_2_ and a 10-fold difference between realgar (As_4_S_4_) and NaAsO_2_. This study used 6 different mammalian cell lines, and the trends are quite similar [92].

### 5.2. Acute and Subacute Toxicity Study in Animals

Lauha Bhasma is a complex herbo-metallic preparation widely used as an Ayurvedic hematinic agent and it was prepared by Ayurvedic procedures of purification (śodhana) and sun drying (bhānupāka), followed by repeated calcination (māraṇa). The resultant product was subjected to acute and subacute toxicity studies. In the acute toxicity study, the animal group did not manifest any signs of toxicity and no mortality was observed up to 100 times the therapeutic dose. Subacutely, it was safe at 5TD dose levels. However, alteration in some of the biochemical and hematological parameters along with histopathological findings were evident at the 10 TD [93]. In subacute toxicity studies, rats were given a Ayurvedic polyherbal medicine* Arogyavardhini Vati* at doses of 50, 250 and 500 mg/kg (1, 5 and 10 times of clinical dose) or HgCl_2_ (1 mg/kg) for 28 days. HgCl_2_ at 1 mg/kg dose produced neurological abnormalities and liver and kidney injuries, while in* Arogyavardhini Vati*-treated mice, even at 500 mg/kg, no toxicity manifestation is overt. Hg accumulation after HgCl_2_ administration in brain, liver and kidney was 12, 27, and 28-fold higher than that after* Arogyavardhini Vati*, respectively [59]. In a subacute study in rats, cinnabar was given at a dose of 1 g/kg and compared to MeHg at a dose of 2 mg/kg for 14 days, followed by a 14-d recovery period. Tissue Hg accumulation increased in both groups but much higher in MeHg group. Animal body weight loss, reduced nerve conduction velocity, reduced rotarod activity, and increased tail flick latency was evident in MeHg group, but unchanged in cinnabar group; After 14-days recovery, Body weight loss and time stay on the rotarod were still lower in MeHg-treated rats, despite a 5000-fold difference in dose levels [95]. Kumar and Gupta [94] further examined the neurotoxicity of three Bhasma-containing Ayurvedic medicines (Calcury, Energic-31, and* Basanta Kusumakara Rasa*) in rats, with the highest dose being 10 times clinical dose (up to 1500 mg/kg) for 28 days, and did not detect neurological abnormalities, either from behavioral, biological (GSH and MDA), and histopathological examination. In comparison, Pb(CH_3_COO)_2_, HgCl_2_, CdCl_2_, and NaAsO_2_ at 10 mg/kg produced significant toxicities with tremendous metal accumulation in brain as compared to Ayurvedic medicines: Hg, 100-fold versus 10-fold, Pb, 260-fold versus 15-fold, As, 350-fold versus 2-fold, and Cd, 650-fold versus 2-fold. Thus, Bhasmas are different from environmental metals in their toxicity and risk.

### 5.3. Subchronic and Chronic Toxicity Studies in Animals

To determine the role of* Shodhana* process in Tamrabhasma preparation, rats were given* Shodhita Tamra* (purified) and* Ashodhita Tamra* (unpurified) at three doses for 45 days, and serology and histopathology were examined.* Shodhita Tamra* is safer than* Ashodhita Tamra*, indicating the importance of* Shodhana* in Bhasma preparation [38]. In another study, mice were given orally Trivanga Bhasma (Pb, Sn and Zn) at 1, 5, and 10 TED dose daily for 90 days, and no overt abnormal changes in the body weight, feed and water consumption, hematology, and histopathological parameters were observed [96]. In mice chronically dosed with the Tibetan medicine Zuotai for 135 days at the clinical dose, no abnormality in behavior and overt histopathology occurred. The results suggest that the clinical dose of Zuotai is safe [97].

### 5.4. Toxicity towards Young and Old Animals

Metal toxicity in vulnerable populations is affected by development, sex, and aging. Mice were exposed to cinnabar (10 mg/kg), HgCl_2_ (0.5 mg/kg), or MeHg (0.02 mg/kg), during the gestation (21-d), lactation (21-d), and/or maturation (7 weeks) and developmental toxicity and neurotoxicity were examined. Long-term exposure to cinnabar did produce Hg accumulation in tissues, retard animal growth, and produce neurobehavioral impairments. However, HgCl_2_ produced more profound changes, and MeHg is much more detrimental, even at 1/200 dose of cinnabar [98]. Similarly, in a study comparing Hg toxicity weanling young rats with aged rats receiving Zuotai, HgS, HgCl_2_ at equivalent Hg doses, and MeHg at 1/10 doses for 7 days, more liver and kidney toxicity was seen in HgCl_2_ and MeHg-treated animals than Zuotai and HgS [99].

Thus, it is apparent that the processed metals in traditional medicines are quite different from metals or metalloids found in our environment, in terms of pharmacokinetics (tissue accumulation), therapeutic effects (brain serotonin levels), and toxicity effects (brain, liver, and kidney). Such differences appear to be dependent on the amount of free metals delivered to the body, but they also depend on biological responses. Because of these huge differences, the experimental design of including environmental metals for comparison could be a strategy to assess the efficacy and risk of metals used in traditional medicines.

## 6. Research Needs and Perspectives

**Figure 1:**
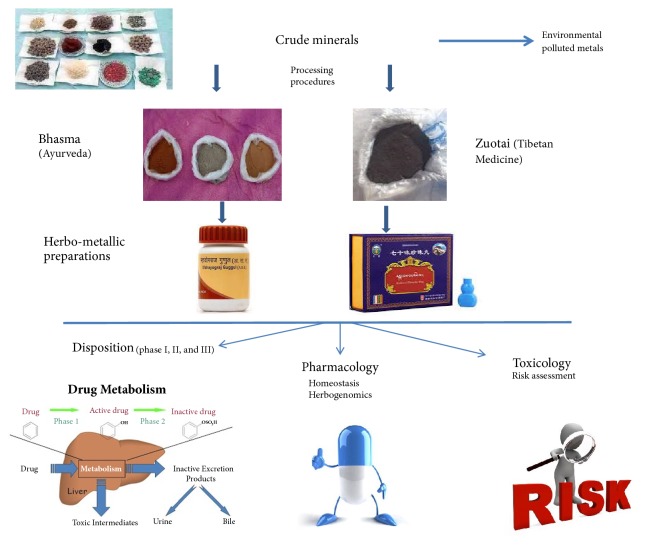
An illustration of herb-metallic preparations and the research needs.

Figure 1 is a graphic summary of this minireview indicating that metals used in traditional medicines are in chemical forms different from environmental metals, and the herbo-metallic preparations rather than metals alone are used in traditional medicines. Future works on their disposition, pharmacology and toxicology are needed to elucidate their benefits and risks.

### 6.1. Processed Bhasmas Differ from Environmental Metal Contaminants

In Bhasmas and other traditional medicines, minerals are subjected to “alchemy” procedures to alter their original forms suitable for medication (i.e., HgS, As_4_S_4_, PbS, PbO), which are distinct from other environmental metal contaminants (HgCl_2_, MeHg, NaAsO_2_, NaH_2_AsO_4_, Pb(CH_3_COO)_2_, etc.). Unprocessed minerals are seldom used in traditional oral remedies. To speciation and identify the chemical forms of minerals/metals used in traditional remedies are critical in evaluating their toxic potentials and potential therapeutics. The allowable metal limits of FDA or EPA are based on metals in the environment. For example, one cannot use the criteria for HgCl_2_ to assess HgS.

### 6.2. Clinical Efficacy and Safety

The use of traditional medicine should be limited for refractory diseases, difficult and complicated diseases such as cancer, stroke, and diabetes as complementary and alternative approaches. In this regard, the clinical efficacy is of utmost importance, and the tolerance is secondary. Ayurveda emphasizes that the drugs should be selected on the basis of individual variations [100].

### 6.3. Study of Herbo-Metallic Preparations

Mixtures differ from individual components in that they could achieve a better efficacy or reduce the toxicity. Herbo-metallic interactions are presumed to assist each other on therapeutics and reduce the toxicity. In this regard, researchers should take mixtures as a whole in intact animals with the traditional oral route of administration to evaluate its action before dissecting the components. For example, HgS and As_4_S_4_ containing An-Gong-Niu-Huang Wan protects against the hepatorenal toxicity of HgS and As_4_S_4_ when minerals were used alone [101].

### 6.4. Metal-Herb-Drug Interactions

As the complementary remedy, traditional medicines are usually used in combination with standard therapeutics. In this regard, any potential effects of Zuotai or Bhasmas on drug metabolizing enzymes, such as phase-1 (e.g., P450), phase 2 (e.g., UDP-glucuronosyltransferase), and phase-3 (uptake and efflux transporters), should be considered [102].

### 6.5. Potential Adverse Effects on the Development and Children

Development and childhood are the vulnerable period to metal toxicity. For the safety of the next generation, any potential adverse effects should not only devote to adults, but also to the susceptible population.

### 6.6. A Roadmap to Address Efficacy and Safety

The National Pharmacovigilance Programme for Ayurveda, Siddha, Unani, Tibetan, and Chinese medicines have been proposed in India [103] and in China [11] to address the efficacy and safety, including the regulation laws, the consumer guidelines, prescription guidelines, clinical monitoring guidelines, and implementation of good manufacturing guidelines [74, 103, 104]. Clinical efficacy is of utmost importance as exemplified by the use of arsenic sulfide in combination with other drugs in the treatment of acute promyelocytic leukemia [90]. Pharmacovigilance is also important to ensure the safety as exemplified by experience in an Ayurvedic Teaching Hospital ([75]), and in patients taken Tibetan medicines [67, 72, 73].

## 7. Conclusions

Chemical forms of metals in Bhasma and Zuotai are a major determinant of their disposition, efficacy and toxicity. The use of total metals for risk assessment is inadequate. Pb, Hg, and As poisoning cases from the use of mineral-containing traditional medicines are mainly due to inappropriate use (high dose and long term administration) and inappropriate preparation procedures. The therapeutic effects of minerals often go together with toxicity. Appropriate evaluation and balance of their efficacy and toxicity based on individuals are important.

## Figures and Tables

**Table 1:** Examples of preparation and analysis of metals/metalloids in traditional medicines.

**Traditional medicine**	**Major metals & chemical forms**	**Other metal/elements**	**References**
***Ayurvedic Medicine***			
Rasasindura	HgS		Kamath et al., 2012 [4]
Makardhwaja	HgS, Au		Khedekar et al., 2011 [21]
Naga Bhasma	PbS, PbO	Si, Fe, K+	Nagarajan et al., 2014 [3]
Tamra Bhasma	CuS	Fe, Mn, Zn, Pb, As, Cd	Jagtap et al., 2012 [22]
Lauha Kalpas	Fe_2_O_3_	Si, Al, Ca	Gupta et al., 2012 [23]
Tarakeshwara Rasa	Fe_2_O_3_	SnO_2_, HgS, SiO_2_, HgO	Virupaksha & Kumar, 2012 [24]
Trivanga Bhasma	Sn, Zn, Pb	Hg, Cu, S, Mica	Rasheed et al., 2014 [25]
Rajata Bhasma	Ag nanoparticles		Sharma et al., 2016 [26]
Yashada Bhasma	Zn, ZnO		Umani et al., 2015 [19]
Jasad Bhasma	ZnO, ZnS,	HgO	Chavare et al., 2017 [27]
Vanga bhasma	SnO_2_		Kale and Rajurkar, 2018 [28]
Praval bhasma	CaO		Mishra et al., 2014 [29]
Mica Ash	Silicate minerals		Wijenayakea et al., 2014 [30]

***Tibetan Medicine***			
Zuotai (Tsothel)	HgS	Fe, Cu, Si, Mg, Ca, Se, K+	Li et al., 2016 [31]
Nengchi Bakuang	K(Mg,Fe)_3_(Al,Fe)Si_3_O_10_(OH, F)_2_	F, S, Cu, Zn, As, Sn, Ca, P, K	Li et al., 2011 [32]
	KMg_3_Si_3_AlO_10_ (F, OH)_2_, Mg_2_SiO_4_, FeS		
Nengchi Bajin	AuPb_2_, Ag_2_S_2_, PbO, CuS,	PbSO_4_, NaCu_2_S_2_, CaCo_3_,	Li et al., 2012 [33]
	SiO_2_, SnS, ZnS	Cu_7_S_4_, CaFe_2_MgC0_3_	

**Table 2:** Comparison of frequently used *β*-HgS and *α*-HgS in traditional medicines.

	**α** **-HgS**	**β** **-HgS**
English name	Cinnabar	Metacinnabar
Element valence state	Hg^2+^, S^2-^	Hg^2+^, S^2-^
Pressing procedure	grinding and washing	Repeated incinaration
Product color	Red	Black
Solubility	Insoluble sulfide	Insoluble sulfide
Bioaccessibility	Poor	Poor
Metabolites	mercuric polysulfide	mercuric polysulfide
TCM name	Zhusha	Zuotai (gTsothal)
In Tibetan and Chinese medicine	*An-Gong-Niu-Huang*	*Rannasangpei (70W)*
In Ayurvedic medicine	*Mahayograj Guggulu*	*Arogyavardhini Bati*

**Table 3:** Examples of evaluation of toxicity in metal-containing traditional medicines.

**Traditional Medicine**	**Experimental model**	**End points**	**Metal toxicity**	**Ref**
***Cytotoxicity***				
HgS, HgCl_2_, MeHg, As^3+^, As^5+^	Cell cultures	Cytotoxciity	HgS < < HgCl_2_ < MeHg	[92]
		MTT assay	As_4_S_4_< < As^3+^< As^5+^	

***Acute and subacute toxicity***				
Lauha Bhasma	Acute 14-d study in rats	Mortality	No acute mortality up to 100 TD	[93]
Arogyavardhini vati	28-d study in rats	Neurotoxicity	Arogyavardhini < HgCl_2_	[59]
		Liver and kidney toxicity, GSH, MDA	Arogyavardhini vati < HgCl_2_	
Energic-31 capsule	28-d study in rats	Learning and memory function	Energic-31 < < HgCl_2_, Pb, Cd, As	[94]
		Brain MDA and GSH		
		Brain metal accumulation		
Calcury tablet	28-d study in rats		Calcury < < HgCl_2_, Pb, Cd, As	
				
Cinnabar (HgS)	14-d study in rats	Neurotoxicity, Na+/K+ ATPase	Cinnabar (1 g/kg) << MeHg (2 mg/kg)	[95]
		Hg accumulation		

***Sub-chronic and chronic toxicity***				
Tamra (copper) bhasma	45-d study in rats	Serology and histopathology	Shodhita Tamra < Ashodhita Tamra	[29]
Trivanga Bhasma	90-d study in mice	Serology and histopathology	No overt injury at 1, 5, 10 TD	[96]
				
Zuotai (gTso thal)	135-d study in mice	No abnormality in serology and morphology		[97]
		Hg accumulation increased but reversible		

***Studies in young and old animals***				
Cinnabar	Weanling nice (7 weeks)	Developmental and neuro-toxicity	Cinnabar << HgCl_2_ or MeHg	[98]
Zuotai (gTso thal)	Weanling and old mice (7-d)	Old mice is more susceptible to Hg toxicity and transporter alterations	Zuotai << HgCl_2_, MeHg	[99]

## Abbreviations

AFM: Atomic force microscopy
ICP-MS: Inductively coupled plasma mass spectrometry
FTIR: Fourier transform infrared spectroscopy
SEM-EDX: Scanning electron microscopy with energy dispersive X-ray spectroscopy
TEM: Transmission electron microscopy
XRD: X-ray diffraction
XAS: X-ray absorption spectroscopy
XANES: X-ray Absorption Near Edge Structure
GAE-AFS: Gold amalgam enrichment-atomic fluorescence spectrometry
FOREhST: Fed Organic Estimation human Simulation Test.
